# MicroRNA Profiling of Pericardial Fluid Samples from Patients with Heart Failure

**DOI:** 10.1371/journal.pone.0119646

**Published:** 2015-03-12

**Authors:** Suvi M. Kuosmanen, Juha Hartikainen, Mikko Hippeläinen, Hannu Kokki, Anna-Liisa Levonen, Pasi Tavi

**Affiliations:** 1 Department of Biotechnology and Molecular Medicine, A. I. Virtanen Institute for Molecular Sciences, University of Eastern Finland, Kuopio, Finland; 2 School of Medicine, University of Eastern Finland, Kuopio, Finland; 3 Heart Center, Kuopio University Hospital, Kuopio, Finland; 4 Anesthesia and Operative Services, Kuopio University Hospital, Kuopio, Finland; University of Colorado Denver, UNITED STATES

## Abstract

**Aims:**

Multicellular organisms maintain vital functions through intercellular communication. Release of extracellular vesicles that carry signals to even distant target organs is one way of accomplishing this communication. MicroRNAs can also be secreted from the cells in exosomes and act as paracrine signalling molecules. In addition, microRNAs have been implicated in the pathogenesis of a large number of diseases, including cardiovascular diseases, and are considered as promising candidate biomarkers due to their relative stability and easy quantification from clinical samples. Pericardial fluid contains hormones secreted by the heart and is known to reflect the cardiac function. In this study, we sought to investigate whether pericardial fluid contains microRNAs and if so, whether they could be used to distinguish between different cardiovascular pathologies and disease stages.

**Methods and Results:**

Pericardial fluid was collected from heart failure patients during open-heart surgery. MicroRNA profiles of altogether 51 patients were measured by quantitative real-time PCR (qPCR) using Exiqon human panels I and II. On the average, 256 microRNAs were detected per sample, and 70 microRNAs out of 742 profiled microRNAs were detected in every sample. The five most abundant microRNAs in pericardial fluid were miR-21-5p, miR-451a, miR-125b-5p, let-7b-5p and miR-16-5p. No specific signatures for cardiovascular pathologies or clinically assessed heart failure stages could be detected from the profiles and, overall, microRNA profiles of the samples were found to be very similar despite the heterogeneity in the study population.

**Conclusion:**

Measured microRNA profiles did not separate the samples according to the clinical features of the patients. However, several previously identified heart failure marker microRNAs were detected. The pericardial fluid microRNA profile appeared to be a result of an active and selective secretory process indicating that microRNAs may act as paracrine signalling factors by mediating the local crosstalk between cardiac cells.

## Introduction

Heart failure (HF) is a condition in which the heart is unable to sustain sufficient blood circulation to meet the needs of the body. Heart failure can result from many cardiovascular conditions, but the most common causes for the development of HF are coronary artery disease, hypertension, and valvular heart diseases. Several pathogenic mechanisms have been found to contribute to disease progression (reviewed in ref [1]). As a consequence, substances are released to the blood stream where they can be detected and used as biomarkers to aid diagnostics and treatment. Of the currently used biomarkers, myocyte stretch-induced natriuretic peptides, especially B-type natriuretic peptide (BNP) and biologically inert but more stable N-terminal proBNP (NT-proBNP), are well validated and widely used in clinical diagnostics [2].

Cardiac tissue produces many physiologically active substances such as cytokines, growth factors and cardiac hormones. Many of these function locally in either an autocrine or paracrine manner in the heart and can be found in high concentrations within pericardial fluid, formed by the active secretion of the pericardial cells and, in part, as a heart tissue filtrate [3]. For example, the concentrations for heart-specific hormones, such as atrial natriuretic peptide (ANP), BNP and endothelin-1 (ET-1), are considerably higher in pericardial fluid compared to plasma [4,5]. In addition, pathological conditions influence the cardiac hormone and growth factor composition of the fluid [6–9]. Pericardial fluid can impact cardiomyocyte growth [10–12] and it appears to contain unidentified components that stimulate cardiac stem cell differentiation into cardiac cells after myocardial infarction [13].

MicroRNAs (miRNAs) are small, non-coding RNAs that control gene expression by inhibiting target messenger RNA translation or increasing mRNA decay [14]. MicroRNAs are important post-transcriptional regulators of most cellular and developmental processes and they have an emerging important role in cardiovascular pathologies, including HF [15–17]. These small RNAs can be secreted in either protein-bound or vesicle-enclosed forms from the cells into the extracellular space or to the systemic circulation where they act as paracrine or endocrine signalling molecules. These circulating miRNAs are considered as candidates for use as biomarkers in molecular diagnostics and therapy [18]. Exosomes are small (30–100 nm) intercellular signalling vesicles that have been shown to carry miRNAs. Cardiac cells release and uptake exosomes and the signalling rate increases under stressful conditions. Exosome signalling is involved in the central processes of cardiac remodelling, and recent reports suggest that cardiac exosomes released after ischemic insult reprogram bone marrow cells to initiate cardiac repair processes [19–24].

Given that cardiomyocytes actively secrete miRNAs and that cardiomyocyte-derived miRNAs can be found in circulation, we hypothesized that these could also be detected in the pericardial fluid, where they may serve as paracrine signalling mediators. In the present study we sought to investigate if pericardial fluid contains miRNAs and whether different cardiac diseases and disease states could be differentiated by the miRNA signatures found in the pericardial fluid.

## Methods

### Ethics statement

The study was approved by the Research Ethics Committee of the Hospital District of Northern Savo, Kuopio, Finland (Permit: 30/2012) and written informed consent was obtained from each participant.

### Study population

All patients were enrolled at Kuopio University Hospital between October 10, 2012 and November 15, 2013. The study included 51 HF patients who were classified into groups according to clinical evaluation. Clinical data for the study population is summarized in Table 1. Patients with coronary artery disease were enrolled in group 1 (*n* = 18), patients with mitral valve insufficiency in group 2 (*n* = 13), patients with aortic stenosis in group 3 (*n* = 4), patients with aortic valve insufficiency in group 4 (*n* = 1), and patients with other cardiovascular disease (CVD) in group 5 (*n* = 5). Patients belonging to more than one of the groups were enrolled in groups 6 to 11 according to their clinical status (6 = Coronary artery disease, mitral insufficiency and other CVD, *n* = 1; 7 = Coronary artery disease and aortic stenosis, *n* = 5; 8 = Mitral insufficiency and aortic stenosis, *n* = 1; 9 = mitral insufficiency, aortic insufficiency and other CVD, *n* = 1; 10 = Mitral insufficiency and other CVD, *n* = 1; 11 = Aortic stenosis and aortic insufficiency, *n* = 1). Patients groups are summarized in Table 2. Patients were also grouped according to the NYHA classification (New York Heart Association functional classification) for the extent of HF: patients without clinical HF belong to group 0 (*n* = 3), patients with cardiac disease but no symptoms and no limitations in ordinary physical activity in group I (*n* = 4), patients with mild symptoms in group II (*n* = 19), patients with marked limitation in activity due to symptoms in group III (*n* = 13), and patients with severe limitations and having symptoms even while at rest in group IV (*n* = 12).

**Table 1:** Clinical characteristics.

Variable	Patients (n = 51)
Gender (male)	37 (73%)
Age (years)*	63.8 ± 9.3
Smoking	8 (16%)
Body mass index*	27.6 ± 4.2
Diabetes mellitus	8 (16%)
Hypertension	29 (57%)
Coronary artery disease	24 (47%)
Mitral insufficiency	17 (33%)
Aortic insufficiency	3 (6%)
Aortic stenosis	11 (22%)
Other cardiovascular disease	8 (16%)
NYHA class grade	
Class 0	3 (6%)
Class I	4 (8%)
Class II	19 (37%)
Class III	13 (25%)
Class IV	12 (24%)

NYHA class = New York Heart Association class

* Mean ± SD.

**Table 2:** Cardiovascular disease groups.

Group	Disease	*n*
1	Coronary artery disease	18
2	Mitral valve insufficiency	13
3	Aortic stenosis	4
4	Aortic valve insufficiency	1
5	Other cardiovascular disease (CVD)	5
6	Coronary artery disease, mitral insufficiency and other CVD	1
7	Coronary artery disease and aortic stenosis	5
8	Mitral insufficiency and aortic stenosis	1
9	Mitral insufficiency, aortic insufficiency and other CVD	1
10	Mitral insufficiency and other CVD	1
11	Aortic stenosis and aortic insufficiency	1

### Sample collection and preparation

Pericardial fluid samples (2–27 ml) were collected during open-heart surgery. Samples were processed directly after sample collection using three-step centrifugation, except those with visible blood contamination that were discarded. Samples were first centrifuged at 300 *g* for 10 min at RT to remove cells. Subsequently the supernatants were collected and then centrifuged again at 16 500 *g* for 20 min at 4°C to remove cell debris. The collected liquid fractions were centrifuged a final time at 20 000 g for 15 min at 4°C to remove other microparticles, leaving exosomes and protein-bound miRNAs to the supernatants. The supernatants were transferred to RNase-free tubes, snap-frozen with liquid nitrogen and stored at -80°C.

### Extended qPCR quality control analysis

RNA isolations and real-time qPCR experiments were performed at Exiqon Services, Vedbaek, Denmark. Total RNA was extracted from 200 μl of triple centrifuged pericardial fluid supernatant using the Qiagen miRNeasy Mini Kit with 1.25 μg/mL of MS2 bacteriophage RNA as a carrier and RNA spike-in controls. RNA was eluted with 50 μL of RNase-free water. 1, 2 and 4 μL RNA was reverse transcribed (RT) in 10 μL reactions using the miRCURY LNA Universal RT microRNA PCR, Polyadenylation and cDNA synthesis kit (Exiqon). Each RT was performed in duplicates, including an artificial RNA spike-in (UniSp6). cDNA was diluted 50 x and assayed in 10 μL PCR reactions according to the protocol for miRCURY LNA Universal RT microRNA PCR; each microRNA was assayed once by qPCR using assays for miR-103, miR-191, miR-23a, miR-30c, miR-451 and RNA spike-ins. Negative controls excluding template from the reverse transcription reaction were performed and profiled like the samples. The amplification was performed in a LightCycler 480 Real-Time PCR System (Roche, Basel, Switzerland) in 384 well plates. The amplification curves were analysed using the Roche LC software, both for determination of Cp (by the second derivative method) and for melting curve analysis. An average Cp was calculated for the duplicate RT’s and evaluation of expression levels was performed based on raw Cp-values.

### RNA extraction, reverse transcription and microRNA real-time qPCR

Total RNA was extracted from triple centrifuged pericardial fluid samples (200 μL) using the miRCURY RNA isolation kit for biofluids (Exiqon). Captured RNA was eluted in 50 μL of RNase free H_2_O. 15 μL of this RNA was reverse transcribed in 75 μL reactions using the miRCURY LNA Universal RT microRNA PCR, Polyadenylation and cDNA synthesis kit (Exiqon). The resulting cDNA was diluted 50 fold in RNase free water and assayed in 10 μL PCR reactions according to the protocol for miRCURY LNA Universal RT microRNA PCR. Each of the 742 microRNAs was assayed once by qPCR on the microRNA Ready-to-Use PCR, Human panel I and panel II using ExiLENT SYBR Green mastermix. Negative controls excluding template from the reverse transcription reaction were performed and profiled like the samples. The amplification was performed in a LightCycler 480 Real-Time PCR System (Roche) in 384 well plates. The amplification curves were analysed using the Roche LC software, both for determination of Cp (by the second derivative method) and for melting curve analysis. The amplification efficiency was calculated using algorithms similar to the LinReg software. All assays were inspected for distinct melting curves and the Tm was checked to be within known specifications for the assay. Furthermore, assays had to be detected with 5 Cp’s less than the negative control and with Cp<37 to be included in the data analysis. Data that did not pass these criteria was omitted from any further analysis. Using NormFinder the best normalizer was found to be the average of assays detected in all samples. All data was normalized to the average of assays detected in all samples (average Cp—assay Cp).

### MicroRNA profiling

Pericardial fluid samples were profiled in two separate projects. First profiling included the pericardial fluid samples from 15 patients and included an extended qPCR quality control analysis of microRNAs. All samples passed the quality control analysis and no signs of PCR reaction inhibition or haemolysis were observed. The expression levels were comparable for all samples included in the analysis, and within the detection limit of the system. The second profiling included samples from 36 patients. Both sets of samples were profiled for microRNAs using Exiqon miRNA PCR Human panels I and II (v2). Sampling, RNA extraction and profiling were performed reproducibly in the two sets and no batch effect was seen from the two rounds of profiling. Therefore, the two data sets were combined and analysed as one.

### Data analyses

The data was analysed using the GenEx software 6.0 (MultiD Analyses AB, Göteborg, Sweden) and GraphPad Prism Software. Statistical significance between different disease or NYHA groups was evaluated with ANOVA using Benjamini-Hochberg correction for multiple testing. Results were considered significant for p<0.05. Heatmaps were generated by using heatmap.2 in the R package gplots and principal component analysis by using GenEx software.

## Results

### MicroRNAs in pericardial fluid

In order to investigate whether pericardial fluid contains microRNAs, 742 miRNAs were measured from the pericardial fluid samples. On the average, 256 miRNAs were detected per sample, the number ranging between 154 and 346 miRNAs per sample. 70 miRNAs were detected in all samples (Table 3) and 17 of them were among the 50 most abundant miRNAs in pericardial fluid (Table 4). The five most abundant microRNAs in pericardial fluid were miR-21–5p, miR-451a, miR-125b-5p, let-7b-5p and miR-16–5p (present in 98–100% of the samples).

**Table 3:** MicroRNAs found in all pericardial fluid samples.

hsa-miR-141–3p	hsa-miR-199a-3p	hsa-miR-143–3p	hsa-miR-26b-5p	hsa-let-7f-5p	hsa-miR-488–3p
hsa-miR-331–3p	hsa-miR-374b-5p	hsa-miR-424–5p	hsa-miR-32–5p	hsa-miR-21–5p	hsa-miR-103a-3p
hsa-miR-30e-5p	hsa-miR-497–5p	hsa-miR-335–5p	hsa-miR-452–5p	hsa-miR-130a-3p	hsa-miR-342–3p
hsa-miR-132–3p	hsa-miR-30c-5p	hsa-miR-10a-5p	hsa-miR-128	hsa-miR-551a	hsa-miR-532–3p
hsa-miR-107	hsa-miR-210	hsa-miR-16–5p	hsa-miR-30a-5p	hsa-miR-29c-5p	hsa-miR-34a-5p
hsa-miR-185–5p	hsa-miR-502–3p	hsa-let-7g-5p	hsa-miR-660–5p	hsa-miR-200a-3p	hsa-miR-532–5p
hsa-miR-22–5p	hsa-miR-29b-3p	hsa-miR-20a-5p	hsa-miR-218–5p	hsa-miR-19b-3p	hsa-miR-30d-5p
hsa-miR-99b-5p	hsa-miR-92a-3p	hsa-let-7i-5p	hsa-miR-590–5p	hsa-miR-23b-3p	hsa-miR-221–3p
hsa-miR-23a-3p	hsa-miR-101–3p	hsa-miR-378a-3p	hsa-miR-423–3p	hsa-miR-140–5p	hsa-miR-15a-5p
hsa-miR-320a	hsa-miR-24–3p	hsa-miR-151a-3p	hsa-miR-186–5p	hsa-miR-144–3p	hsa-miR-126–3p
hsa-miR-30b-5p	hsa-miR-193b-3p	hsa-miR-99a-3p	hsa-miR-484	hsa-miR-25–3p	hsa-miR-425–5p
hsa-miR-505–3p	hsa-miR-31–3p	hsa-miR-423–5p	hsa-miR-99a-5p		

**Table 4:** MicroRNAs found in all samples among the 50 most abundant microRNAs.

MicroRNA	Amount* (Average Cp- Assay Cp)	Presence (*n* = 51)
**miR-21–5p**	6.4	51 (100%)
**obsolete_ miR-720**	5.3	51 (100%)
**miR-320a**	3.8	51 (100%)
**miR-24–3p**	3.6	51 (100%)
**miR-16–5p**	3.5	51 (100%)
**miR-19b-3p**	3.4	51 (100%)
**miR-15a-5p**	3.3	51 (100%)
**miR-23a-3p**	3.3	51 (100%)
**miR-99a-5p**	2.9	51 (100%)
**miR-101–3p**	2.7	51 (100%)
**miR-34a-5p**	2.3	51 (100%)
**miR-92a-3p**	2.2	51 (100%)
**miR-23b-3p**	2.2	51 (100%)
**miR-378a-3p**	1.6	51 (100%)
**miR-221–3p**	1.5	51 (100%)
**miR-20a-5p**	1.2	51 (100%)
**miR-423–3p**	1.2	51 (100%)

*Average on all samples

MicroRNAs have been detected from all body fluids profiled so far. Therefore, we sought to investigate if any of the most detected miRNAs were specific for the pericardial fluid and, thus, could reflect the miRNA expression of the surrounding tissues. When comparing the miRNAs detected from the pericardial fluid samples over the global mean (i.e. Cp < 30) with the 12 body fluid types profiled previously by Weber and others [25], six of the miRNAs (miR-125b-5p, miR-320b, miR-34a-5p, miR-497–5p, miR-99b-5p and let-7d-3p) were found to be specific for pericardial fluid (Table 5). Seven miRNAs (miR-21–5p, miR-148a-3p, miR-152, miR-93–5p, miR-29b-3p, miR-184 and miR-218–5p) were present in all body fluids.

**Table 5:** MicroRNAs in body fluids.

MicroRNAs specific to PF	Amount* (Average Cp- Assay Cp)	Presence (*n* = 51)
**miR-125b-5p**	5.1	50 (98%)
**miR-320b**	3.5	49 (96%)
**miR-34a-5p**	2.3	51 (100%)
**miR-497–5p**	0.8	51 (100%)
**miR-99b-5p**	0.8	51 (100%)
**let-7d-3p**	0.8	50 (98%)

**MicroRNAs present in all fluids**	Amount* (Average Cp- Assay Cp)	**Presence (*n* = 51)**
**miR-21–5p**	6.4	51 (100%)
**miR-148a-3p**	2.1	46 (90%)
**miR-152**	0.8	50 (98%)
**miR-93–5p**	0.7	50 (98%)
**miR-29b-3p**	0.4	51 (100%)
**miR-184**	0.2	1 (2%)
**miR-218–5p**	0.007	51 (100%)

PF = pericardial fluid

*Average on all samples

### MicroRNA clusters and families

**Fig 1:**
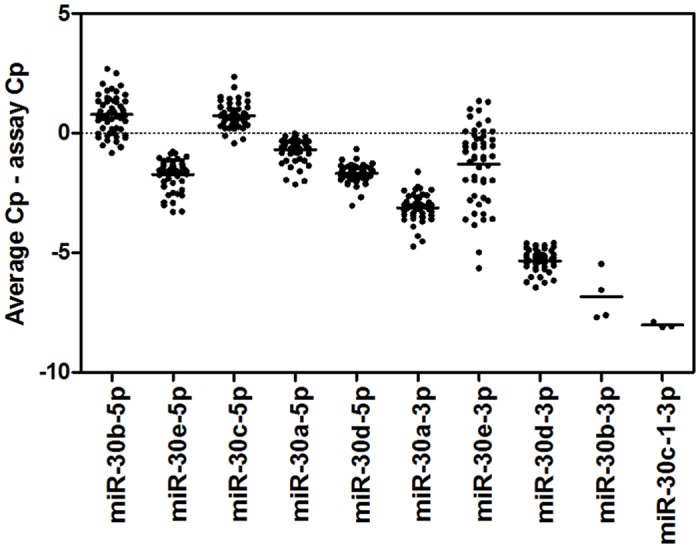
MicroRNA gene family miR-30. The presence of all mir-30 family members in pericardial fluid was investigated using qPCR. Results are depicted as individual points for each measured sample (*n* = 51) lines indicating the mean expression for each miRNA.

MicroRNAs arising from the same genomic loci (<10 kb) are defined as miRNA clusters, whereas miRNA family members share 5’ seed sequences, which establish target specificity [26]. When analysing miRNAs detected in at least 80% of the samples, several miRNA clusters were found to be present and several of the detected miRNAs belonged to the same miRNA family (S1 and S2 Tables). The expression levels for the mir-30 family are shown in Fig. 1. The family members arise from several different genomic loci, both from miRNA clusters and distinct miRNA genes.

### Cardiac microRNAs in pericardial fluid

MiRNAs derived from myocin heavy chain (MHC) genes, which are highly enriched in cardiac and/or skeletal muscle, are called myomiRs. They include miR-1, miR-133, miR-206 (skeletal muscle only), miR-208, miR-486 and miR-499 [27]. MicroRNA-1 (miR-1) is described as being the most abundant miRNA in heart tissue. Pericardial fluid contained only low concentrations of miR-1 and it could be detected in 55% of samples (Table 6). Also miR-133a and miR-133b were measured at low concentrations and only in 45% and 63% of samples, respectively. MiR-208a and miR-208 were recorded in 6% and 4% of samples, respectively. MiR-486–5p was present in 92% of samples despite its complementary strand sequence being undetected. MiR-499a-5p was present in 33% of samples, whereas miR-499a-3p was present in only 2% of samples.

**Table 6:** Cardiac microRNAs in pericardial fluid.

MyomiRs	Amount* (Average Cp- Assay Cp)	Presence (*n* = 51)
**miR-1**	-6.2	28 (55%)
**miR-133a**	-6.6	23 (45%)
**miR-133b**	-5.7	32 (63%)
**miR-208a**	-7.6	3 (6%)
**miR-208b**	-6.7	2 (4%)
**miR-486–5p**	-1.7	47 (92%)
**miR-486–3p**		0 (0%)
**miR-499a-5p**	-7.3	17 (33%)
**miR-499a-3p**	-4.4	1 (2%)

**Fibroblast microRNAs**	Amount* (Average Cp- Assay Cp)	**Presence (*n* = 51)**
**miR-21–5p**	6.4	51 (100%)
**miR-29a-3p**	2.8	50 (98%)
**miR-29b-3p**	0.4	51 (100%)
**miR-29c-3p**	3.4	50 (98%)
**miR-30a-5p**	-0.7	51 (100%)
**miR-30b-5p**	0.8	51 (100%)
**miR-30c-5p**	0.7	51 (100%)
**miR-30d-5p**	-1.7	51 (100%)
**miR-30e-5p**	-1.7	51 (100%)

**Endothelial cell microRNAs**	Amount*(Average Cp- Assay Cp)	**Presence (*n* = 51)**
**miR-126–3p**	1.4	51 (100%)
**miR-17–5p**	-4.2	47 (92%)
**miR-17–3p**	-3.7	50 (98%)
**miR-18a-5p**	-6.5	23 (45%)
**miR-19a-3p**	-1.8	37 (73%)
**miR-19b-3p**	3.4	51 (100%)
**miR-20a-5p**	1.2	51 (100%)
**miR-92a-3p**	2.2	51 (100%)
**miR-23a-3p**	3.3	51 (100%)
**miR-23b-3p**	2.2	51 (100%)
**miR-24–3p**	3.6	51 (100%)
**miR-27a-3p**	3.1	50 (98%)
**miR-27b-3p**	3.5	50 (98%)
**miR-221–3p**	1.5	51 (100%)
**miR-222–3p**	2.2	50 (98%)

*Average on all samples

Cardiac fibrosis disrupts normal myocardial structures and increases mechanical stiffness that in turn leads to contractile dysfunction of the heart. Fibroblasts synthesize the components of extracellular matrix required for fibrosis in both healthy and pathological hearts. MiR-21–5p is expressed in all cardiovascular cell types, but most prominently in cardiac fibroblasts which are the main source of miR-21–5p in failing hearts [28]. In addition to miR-21, members of the miR-29 family (miR-29a/b/c-3p) and miR-30 family (miR-30a/b/c/d/e-5p) are also highly expressed in fibroblasts. In the present data, miR-21–5p was detected in high levels in all the measured samples. The levels of the miRNAs in pericardial fluid are shown in Table 6.

In addition to cardiomyocytes and fibroblasts, the myocardium consists of a large number of endothelial cells that secrete bioactive substances that influence cardiac growth and contractile function [29]. Endothelial cells also contain specific miRNAs that not only regulate important processes such as angiogenesis, but are also involved in intercellular communication affecting adjacent cells [30–32]. Endothelial cell enriched miRNAs include miR-126–3p, miR-17~92 cluster (miR-17–5p/-3p, miR-18a-5p, miR-19a/b-3p, miR-20a-5p, miR-92a-3p), miR-23~27~24 clusters (miR-23a/b-3p, miR-24–3p, miR-27a/b-3p), miR-221–3p and miR-222–3p [28]. In pericardial fluid, miR-126–3p, miR-23~27~24 cluster miRNAs, miR-221–3p and miR-222–3p were present in relatively high amounts, whereas miR-17~92 cluster miRNAs were detected sporadically (Table 6).

### Cardiac exosomal microRNAs

Human cardiovascular cells have been shown to secrete exosomes that transfer information to recipient cells [20–24,33,34]. The most highly enriched miRNAs, found in extracellular vesicles, secreted by cardiac progenitor cells, cardiomyocytes, cardiac fibroblasts and endothelial cells and their amounts in pericardial fluid are listed in Table 7. Most of the known cardiac exosomal miRNAs were present in pericardial fluid and vast majority of them have been reported of being enriched in fibroblast-derived exosomes.

**Table 7:** Cardiac exosomal microRNAs in pericardial fluid.

MicroRNAs	Amount* (Average Cp- Assay Cp)	Presence (*n* = 51)	Fibroblast-derived exosomes
**miR-210**	-0.2	51 (100%)	
**let-7b-3p**	-2.5	47 (92%)	Yes
**let-7d-3p**	0.8	50 (98%)	Yes
**miR-1**	-6.1	28 (55%)	
**miR-125a-5p**	2.0	41 (80%)	Yes
**miR-126–3p**	-1.0	51 (100%)	
**miR-129–5p**	-8.8	1 (2%)	Yes
**miR-132–3p**	-1.5	51 (100%)	Yes
**miR-133a**	-6.6	23 (45%)	Yes
**miR-135a-5p**	-4.5	47 (92%)	Yes
**miR-135b-5p**	-2.9	22 (43%)	Yes
**miR-138–5p**	-4.9	26 (51%)	Yes
**miR-139–5p**	-2.2	47 (92%)	Yes
**miR-140–5p**	-0.6	51 (100%)	Yes
**miR-143–3p**	-1.5	51 (100%)	
**miR-145–5p**	-2.9	50 (98%)	
**miR-146a-3p**	-7.5	3 (6%)	
**miR-146a-5p**	-1.2	46 (90%)	
**miR-17–5p**	-4.2	47 (92%)	Yes
**miR-181a-5p**	-1.2	50 (98%)	Yes
**miR-181b-5p**	-4.5	47 (92%)	Yes
**miR-181c-5p**	-3.6	50 (98%)	Yes
**miR-208a**	-7.6	3 (6%)	
**miR-20a-5p**	1.2	51 (100%)	Yes
**miR-21–3p**	-5.5	38 (75%)	Yes
**miR-214–3p**	-4.8	29 (57%)	Yes
**miR-23a-3p**	3.3	51 (100%)	Yes
**miR-23b-3p**	2.2	51 (100%)	Yes
**miR-25–3p**	-1.1	51 (100%)	Yes
**miR-30a-3p**	-3.1	49 (96%)	Yes
**miR-30c-5p**	0.7	51 (100%)	Yes
**miR-30e-3p**	-2.5	50 (98%)	Yes
**miR-320a**	3.8	51 (100%)	Yes
**miR-330–5p**	-7.4	8 (16%)	Yes
**miR-339–3p**	-4.1	46 (90%)	Yes
**miR-346**	-5.2	19 (37%)	Yes
**miR-34c-3p**	-7	7 (14%)	Yes
**miR-365a-3p**	1.3	50 (98%)	Yes
**miR-375**	-7.5	6 (12%)	Yes
**miR-499a-5p**	-7.3	17 (33%)	
**miR-505–3p**	-2.1	51 (100%)	Yes
**miR-532–3p**	-0.2	51 (100%)	Yes
**miR-671–5p**	-5.7	2 (4%)	Yes
**miR-92b-3p**	-3.4	50 (98%)	Yes
**miR-9–3p**	-7.9	1 (2%)	Yes

*Average on all samples

### Blood cell derived microRNAs

Although frank haemolysis was excluded from the samples by assessing the miR-23a-3p/miR-451a ratio (S1 Fig.), the blood cell origin of the pericardial fluid miRNAs cannot be excluded, especially as pericardial fluid has been reported to contain high number of lymphocytes and monocytes [35]. The inspection of the levels of 40 miRNAs highly expressed in blood cells [36] (Table 8) revealed that although several of the blood cell miRNAs were present in almost all samples, the overall levels of the miRNAs were not strikingly high.

**Table 8:** Blood cell microRNAs in pericardial fluid.

MicroRNA	Amount* (Average Cp- Assay Cp)	Presence (*n* = 51)	Highest Expression
**miR-223–3p**	0.9	50 (98%)	Neutrophils
**miR-16–5p**	3.5	51 (100%)	Red blood cell
**miR-126–3p**	-1.0	51 (100%)	Platelets
**miR-142–3p**	-2.1	49 (96%)	Neutrophils
**miR-21–5p**	6.4	51 (100%)	Monocytes
**miR-24–3p**	3.6	51 (100%)	Neutrophils
**miR-19b-3p**	3.4	51 (100%)	Neutrophils
**miR-103a-3p**	0.08	50 (98%)	Monocytes
**let-7a-5p**	-2.0	50 (98%)	Neutrophils
**miR-451a**	5.0	50 (98%)	Red blood cells
**miR-92a-3p**	2.2	51 (100%)	Red blood cells
**miR-106a-5p**	0.04	50 (98%)	Neutrophils
**miR-19a-3p**	-1.8	37 (73%)	Neutrophils
**miR-30b-5p**	0.8	51 (100%)	Neutrophils
**miR-17–5p**	-4.2	47 (92%)	Neutrophils
**miR-15b-5p**	0.6	50 (98%)	Neutrophils
**miR-107**	-0.6	51 (100%)	Neutrophils
**let-7f-5p**	-1.7	51 (100%)	Monocytes
**miR-221–3p**	1.5	51 (100%)	Platelets
**miR-93–5p**	0.7	50 (98%)	Neutrophils
**miR-30c-5p**	0.7	51 (100%)	Neutrophils
**miR-151a-5p**	-0.1	49 (96%)	Platelets
**miR-30e-5p**	-1.7	51 (100%)	Monocytes
**miR-30d-5p**	-1.7	51 (100%)	Neutrophils
**miR-486–5p**	-1.7	47 (92%)	Red blood cells
**miR-25–3p**	-1.1	51 (100%)	Neutrophils
**miR-181a-5p**	-1.2	50 (98%)	Neutrophils
**miR-146a-5p**	-1.2	46 (90%)	Lymphocytes
**let-7d-5p**	-2.5	47 (92%)	Neutrophils
**miR-197–3p**	-1.9	49 (96%)	Neutrophils
**miR-106b-5p**	-4.2	38 (75%)	Neutrophils
**miR-148b-3p**	0.6	50 (98%)	Neutrophils
**miR-766–3p**	-7.0	15 (29%)	Neutrophils
**miR-20b-5p**	-6.5	20 (39%)	Red blood cells
**miR-328**	-2.3	50 (98%)	Neutrophils
**miR-574–3p**	-0.4	46 (90%)	Monocytes
**miR-155–5p**	-5.9	18 (35%)	Lymphocytes
**miR-140–5p**	-0.6	51 (100%)	Neutrophils
**miR-425–3p**	-3.7	49 (96%)	Neutrophils
**miR-150–5p**	-1.2	49 (96%)	Lymphocytes

*Average on all samples

### MicroRNA expression signatures

**Fig 2:**
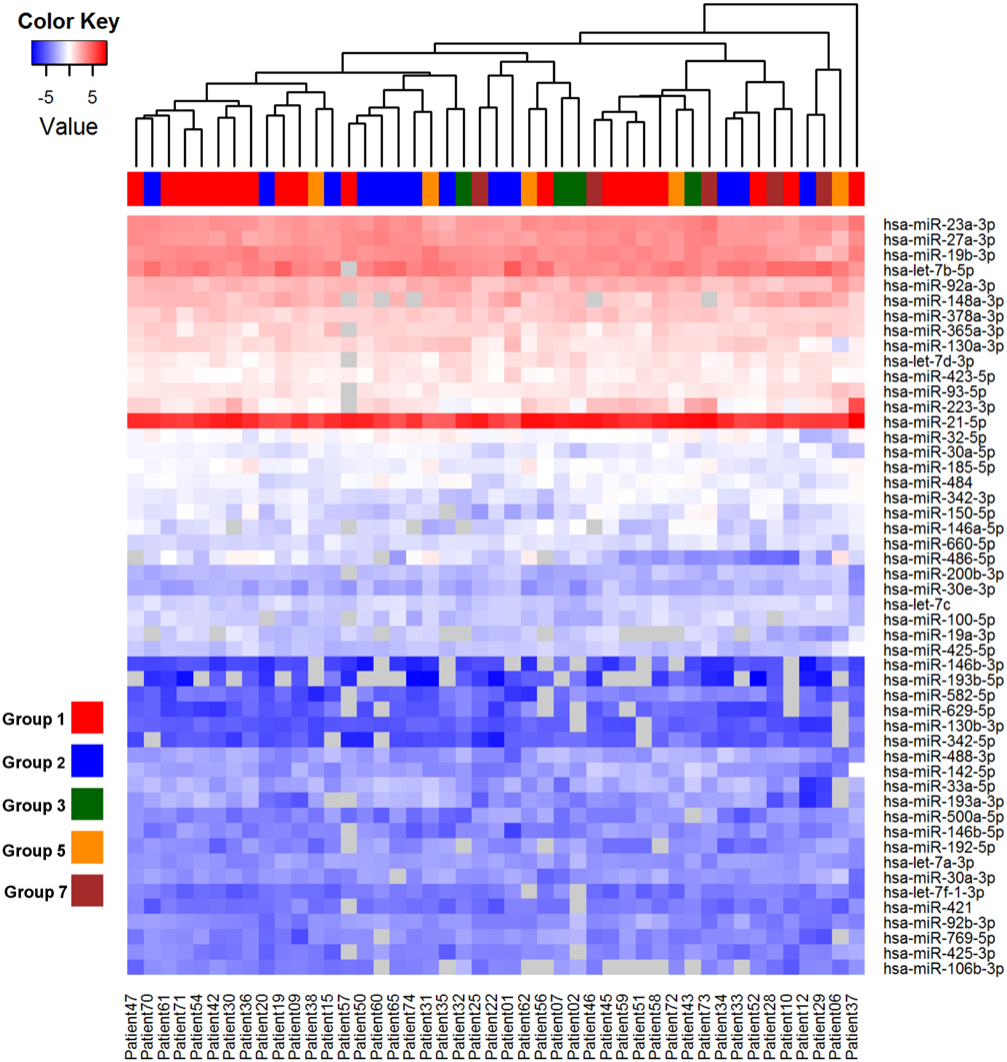
Heat map and unsupervised hierarchical clustering for disease aetiology. The clustering is performed on top 50 miRNAs with highest standard deviation between groups and groups with three or more members. Samples were grouped according to the disease aetiologies of the patients: Group 1: Coronary artery disease (red), Group 2: Mitral valve insufficiency (blue), Group 3: Aortic stenosis (darkgreen), Group 5: Other cardiovascular disease (orange), and Group 7: Coronary artery disease and aortic stenosis (brown). The normalized (dCp) values were used for the analysis. The colour scale illustrates the relative expression level of miRNAs across all samples: red colour represents an expression level above mean, blue colour lower than the mean. Missing values are shown in grey.

**Fig 3:**
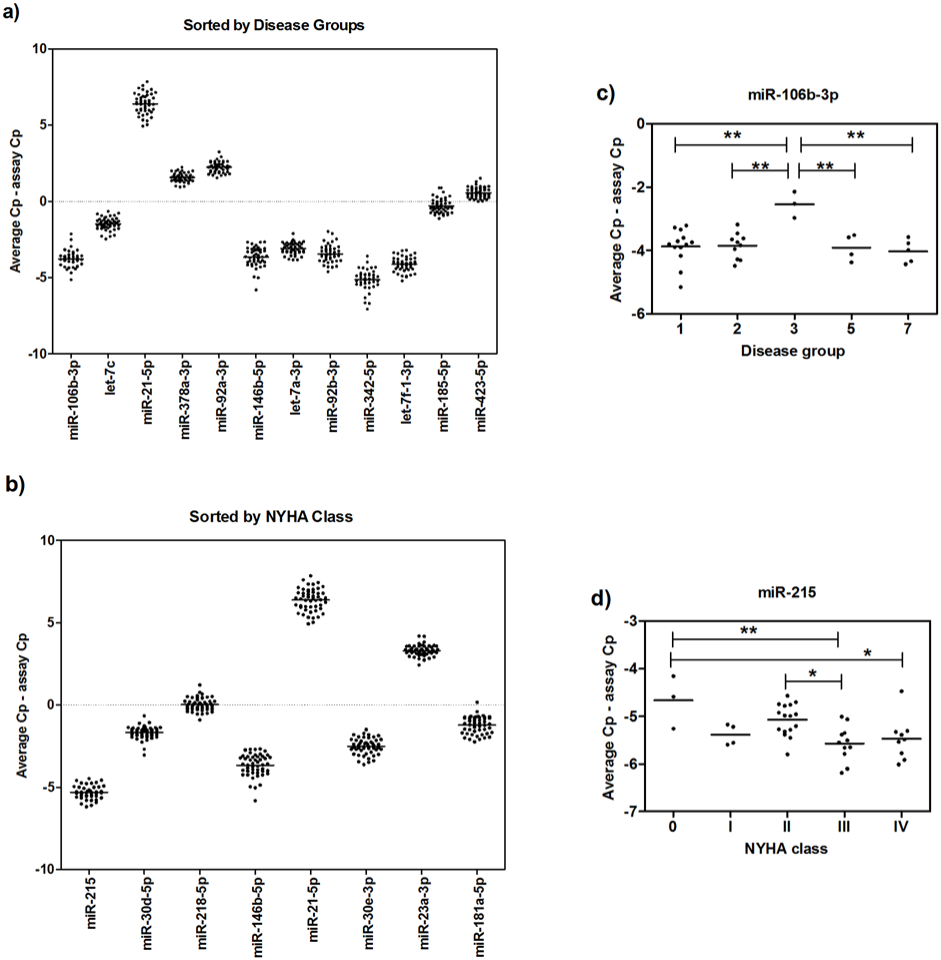
Differentially detected microRNAs by ANOVA. The presence of miRNAs with highest standard deviation between **a)** disease groups (Group 1: Coronary artery disease, Group 2: Mitral valve insufficiency, Group 3: Aortic stenosis, Group 5: Other cardiovascular disease, and Group 7: Coronary artery disease and aortic stenosis) and **b)** NYHA classes were measured using qPCR. Results for **c)** miR-106b-3p and **d)** miR-215 are shown by disease groups and NYHA classes, respectively. Results are depicted as individual points for each measured sample (*n* = 45 for disease, and *n* = 51 for NYHA classes) lines indicating the overall mean for each miRNA. All miRNAs were not detected in every sample. *p<0.05, **p<0.01.

The overall pericardial fluid miRNA profiles of the cardiac patients were similar despite the different disease aetiologies and stages of cardiac dysfunction (S2 Fig.) and no naturally arising sample classes were found using principal component analysis (S3 Fig.). Disease-specific signatures could not be detected from the heatmap even when the analysis was performed on the top 50 miRNAs with highest standard deviation between groups, and unsupervised hierarchical clustering showed that the samples did not cluster according to their clinical groups (Fig. 2). When comparing the groups using ANOVA, 12 miRNAs (miR-106b-3p, let-7c, miR-21–5p, miR-378a-3p, miR-92a-3p, miR-146b-5p, let-7a-3p, miR-92b-3p, miR-342–5p, let-7f-1–3p, miR-185–5p and miR-423–5p) were found to be differentially detected (*p*<0.05) (Figs. 3A and 3C). However, none of these miRs passed the Benjamini-Hochberg correction for multiple testing, failing to reach significance.

**Fig 4:**
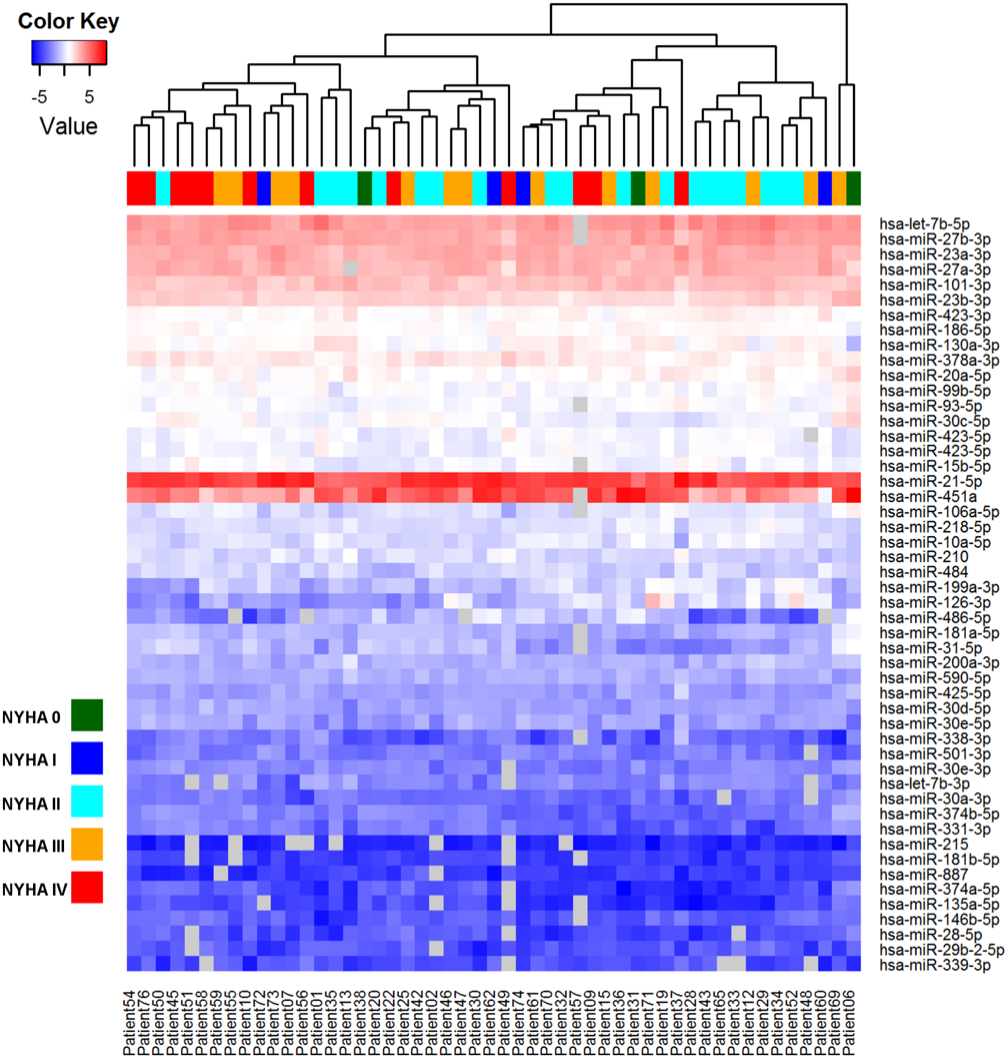
Heat map and unsupervised hierarchical clustering for heart failure stages. The clustering is performed on top 50 miRNAs with highest standard deviation between groups. Samples were grouped according to the NYHA grading of the patients: NYHA 0 (dark green), NYHA I (blue), NYHA II (cyan), NYHA III (orange), NYHA IV (red). Normalized (dCp) values were used for the analysis. The colour scale illustrates the relative expression level of miRNAs across all samples: red colour represents an expression level above mean, blue colour lower than the mean. Missing values are shown in grey.

The data was also analysed based on the assessed stage of HF according to the NYHA class. Patients with no symptoms of HF were categorized to class 0, whereas the most severe clinically assessed HF cases went to class IV. Again, the signatures for the HF stages could not be concluded from the heatmap when using top 50 miRNAs with the highest standard deviation between the groups for the analysis and unsupervised hierarchical clustering failed to cluster the samples according to the stage of the HF (Fig. 4). Eight miRNAs (miR-215, miR-30d-5p, miR-218–5p, miR-146–5p, miR-21–5p, miR-30e-3p, miR-23a-3p and miR-181a-5p) were found to be differentially detected between the NYHA classes (ANOVA, p<0.05), but they failed to pass Benjamini-Hochberg correction for false positivity in multiple testing (Figs. 3B and 3D).

### Heart failure markers in pericardial fluid

**Fig 5:**
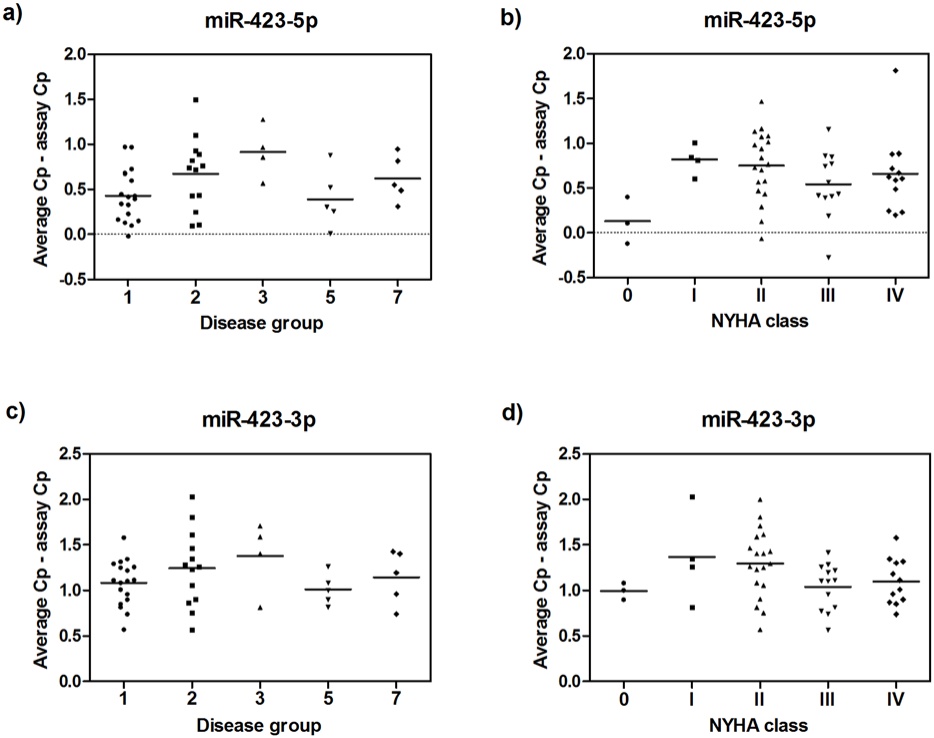
Heart failure marker miR-423–5p in pericardial fluid samples. The presence of miRNAs by **a)** disease groups with three or more members (Group 1: Coronary artery disease, Group 2: Mitral valve insufficiency, Group 3: Aortic stenosis, Group 5: Other cardiovascular disease, and Group 7: Coronary artery disease and aortic stenosis), and **b)** NYHA grading for miR-423–5p and by **c)** disease group and **d)** NYHA grading for miR-423–3p were measured using qPCR. Results are depicted as individual points for each measured sample (*n* = 45 for disease, and *n* = 51 for NYHA classes) lines indicating the overall mean for each group.

One of the most frequently identified circulating miRNAs in HF is miR-423–5p [37–41]. Both miR-423–5p and its counter miRNA, miR-423–3p, were detected in pericardial fluid of the HF patients. MiR-423–3p was detected in all samples and miR-423–5p was detected in 50 out of 51 samples. When samples were divided into groups according to the disease aetiology or NYHA classification, there were no statistically significant differences in the levels of miR-423–5p or miR-423–3p between the groups (Fig. 5). Several other previously suggested HF miRNA markers [17,38] were also detected, but none of them showed significant variation across disease stages (S4 Fig.).

## Discussion

Extracellular miRNAs are extremely stable as they are protected from RNase degradation either by being loaded into small spherical vesicles, such as microvesicles, exosomes, and apoptotic bodies, or by being associated with RNA-binding proteins such as Argonaute2 (Ago2) or lipoproteins such as HDL [20]. Several types of cells, including cardiomyocytes, cardiac fibroblasts and endothelial cells, are known to secrete microRNAs which are taken up by recipient cells, although several questions remain regarding the mechanisms of secretion, targeting, uptake and downstream signalling [21–24]. MiRNAs released from a damaged or diseased organ could potentially act as intercellular communicators and affect the function of distant organs, such as bone marrow [21]. In 2008, the existence of microRNAs in plasma and serum was reported for the first time [42,43]. Since then, several studies have shown that, in addition to plasma and serum, miRNAs are also present in other types of body fluids, such as saliva, tears, cerebrospinal fluid, peritoneal fluid and urine [25]. To our knowledge, this is the first study to explore the pericardial fluid miRNAs.

Pericardial fluid is suggested to form both through active secretion of pericardial cells and as a heart tissue filtrate [3]. Pericardial fluid contains hormones secreted by the heart and reflects the cardiac function more accurately than, for example, plasma obtained from coronary circulation. Therefore, it would be reasonable to assume that the most abundant cardiac miRNAs would be present in the pericardial fluid. MyomiRs (miR-1, miR-133, miR-206, miR-208, miR-486 and miR-499) are highly enriched in cardiac and skeletal muscle, miR-1 being the most abundant miRNA in the heart [44]. However, pericardial fluid contained low concentrations of miR-1 and, moreover, it was detected in only 55% of the samples. In addition, other myomiRs and their complementary strands were detected in low concentrations, if at all, and only miR-486–5p, which has been associated with impaired systemic right ventricular contractility after atrial switch operation for complete transposition of the great arteries [45,46], is measurable in almost all samples (92%). In contrast to myomiRs, pericardial fluid contains significant amounts of both fibroblast and endothelium-enriched miRNAs indicating that these cell types contribute to the composition of the fluid in addition to exosomal miRNAs secreted by the cardiac cells. Pericardial fluid has been reported to contain high lymphocyte and monocyte counts [35], but the inspection of the levels of 40 miRNAs with high blood cell expression revealed that, although the blood cell originated miRNAs most likely affect the miRNA content of the pericardial fluid, they do not predominate the profile. Several of the detected pericardial fluid miRNAs arise from the same genomic locations (cluster miRNAs), and/or belong to a miRNA family, the largest miRNA families detected being let-7, mir-10, mir-30, mir-29, mir-15, mir-17, and mir-181. Taken together, these findings suggest that miRNAs are secreted from cardiac cells through an active and selective process, and that pericardial miRNome is likely to reflect cardiac function.

To highlight the notion that pericardial miRNome reflects the functional state of the myocardium, the five most abundant miRNAs present in pericardial fluid have been associated with cardiovascular disease. These were miR-21–5p, miR-451a, miR-125b-5p, let-7b-5p and miR-16–5p (found in 98–100% of the samples). MiR-21 is highly expressed in cardiovascular system and predominantly expressed in cardiac fibroblasts compared to other cardiac cells [47,48]. Its expression is deregulated in multitude of cardiovascular disease conditions, including HF [49]. In addition, the causal role of miR-21 in fibrosis has been confirmed in heart [50,51], lung [52], kidney [53,54], and skeletal muscle [55]. MiR-451, on the other hand, has been shown to be protective against ischemic damage of the myocardium in several previous studies [56–58] and is upregulated in human hearts after myocardial infarction [59]. Both miR-21 and miR-451 have been identified as potential biomarkers for vulnerable coronary artery disease [60]. Interestingly, miR-21–3p, counterpart of miR-21, acts as a paracrine signalling mediator between fibroblasts and cardiomyocytes and is involved in the development of cardiomyocyte hypertrophy [34]. In addition, the expression level of miR-21–3p was upregulated in pericardial fluid of mice with cardiac hypertrophy compared to sham-operated mice, suggesting that changes in miRNA expression in pericardial fluid indeed provide information about the pathological status of the heart [34]. In addition to miR-21 and miR-451a, miR-125b may also be involved in the pathogenesis of coronary artery disease [61]. Peripheral blood mononuclear cells of the ischemic HF patients [62] as well as plasma from patients with acute myocardial infarction have been shown to contain significantly lower levels of miR-125b compared to control groups [61]. MiR-125b is known to be especially enriched in cardiac valve [63] and has been shown to protect the myocardium from ischemia/reperfusion injury by decreasing infarct size by 60% in addition to preventing decreases in ejection fraction and fractional shortening [64]. Let-7b-5p belongs to the let-7 family which is highly expressed in the cardiovascular system. Aberrant expression of the family members has been shown in several cardiovascular conditions, including cardiac hypertrophy, cardiac fibrosis, myocardial infarction and atherosclerosis [65]. Circulating let-7b levels are lower after acute myocardial infarction in comparison to controls [66] as well as in patients with large-vessel atherosclerosis compared to healthy volunteers [67]. In contrast, circulating levels of miR-16 have been shown to be significantly increased in response to hypertension-induced HF in rats [39].

Comparison of the pericardial fluid miRNA profile to other biofluids revealed that 7 miRNAs (miR-21–5p, miR-148a-3p, miR-152, miR-93–5p, miR-29b-3p, miR-184 and miR-218–5p) were present in all fluid types and 6 miRNAs (miR-125b-5p, miR-320b, miR-34a-5p, miR-497–5p, miR-99b-5p and let-7d-3p) were specific to pericardial fluid. All of the pericardial fluid-specific miRNAs have been previously associated with HF or other cardiovascular conditions. For example, miR-125b, miR-320b, miR34a-5p and miR-497–5p have been associated with acute myocardial infarction [61,68–70] affecting its occurrence, pathogenesis and mortality risk, and miR-125b, miR-320b, miR-497–5p and miR-99b-5p have been associated with atherosclerosis and coronary artery disease development [61,71,72]. In addition, miR-34a-5p, which was detected in high amounts in all pericardial fluid samples, has been identified as a factor contributing to the aging-related decline in cardiac function [73,74]. Whereas miR-99b-5p and members of the let-7 family have been suggested to play a role in endothelial cell differentiation [75] and contribute to the diversity of endothelial cells [76], respectively. Taken together these results suggest that the pericardial miRNA profile is highly cardiac-specific and changes to it may reflect heart pathophysiology.

Although circulating microRNAs possess several of the essential characteristics of a good biomarker [77], verified miRNA signatures for different cardiac conditions have not been identified and consistent miRNA biomarkers for cardiovascular diseases have not been found. In this study, several previously suggested HF markers, such as miR-423–5p, miR-320a and miR-21–5p, were also detected from the pericardial fluid samples but none of them showed significant differences between the different disease aetiologies or stages of the disease. The pericardial fluid samples were obtained from patients undergoing open-heart surgery and, thus, no healthy control samples could be obtained. Therefore, it cannot be concluded from the present data if the miRNA profile resembles normal pericardial fluid or reflects cardiac malfunction. The overall pericardial fluid miRNA profiles were found to be similar independent of the disease background (ischemic vs. nonischemic) or the stage of the HF. This notion is in accordance with a recent publication by Yang and others [78] where they studied myocardial mRNA, miRNA and long noncoding RNA (lncRNA) expression in failing human hearts before and after mechanical support with a left ventricular assist device (LVAD) by using sequencing-based transcriptome profiling. They concluded that the expression profiles of lncRNAs, not mRNAs or miRNAs, were able to discriminate hearts failing from different pathologies as well as between different stages of the same disease as the lncRNA profiles were markedly altered in response to LVAD support.

MicroRNAs are present in body fluids in low amounts and the normalization for miRNA levels is lacking. In addition, subjectivity in the assessment of NYHA classes and the limited size of the study population may mask the small differences arising from the different disease stages and cardiac conditions of the patients. On the other hand, it may be that regardless of the aetiology of the HF, there is a common mechanism for the failure progression and that the changes predicting the development of HF are reflected in the pericardial fluid long before the clinical diagnosis explaining the lack of obvious differences between early and advanced stages of HF.

In summary, human pericardial fluid contains hundreds of miRNAs including several of those that have been previously suggested as HF markers. The overall miRNA profiles of the cardiac patients were similar despite the differences in disease aetiologies and HF stages. Given that the pericardial fluid miRNA profile appeared to be a result of an active and selective secretory process, we surmise that miRNAs may act as endocrine and paracrine signalling factors by mediating the local crosstalk between cardiac cells.

## Supporting Information

S1 Fig Haemolysis. The ratio of two miRNAs is used to monitor haemolysis. MiR-451a is highly expressed in red blood cells, whereas miR-23a-3p is stably expressed and not affected by haemolysis. Samples with ratios above 8.0 are indicative of haemolysis.
(DOCX)

S2 Fig Heat map and unsupervised hierarchical clustering. The clustering was performed on all samples and on the top 50 miRNAs with highest standard deviation. The normalised (dCp) values were used for the analysis. The colour scale illustrates the relative expression level of microRNA across all samples: red colour represents an expression level above mean, blue colour lower than the mean. The sample groups are colour-coded according to their NYHA grading: NYHA 0 (dark green), NYHA I (blue), NYHA II (cyan), NYHA III (orange), NYHA IV (red).
(DOCX)

S3 Fig Principal component analysis. The analysis is performed on all samples and on the top 50 miRNAs with highest standard deviation. The normalized (dCp) values were used for the analysis. Dots represent individual patients and colours indicate NYHA grading: NYHA 0 (green rectangle), NYHA I (blue circle), NYHA II (aqua triangle), NYHA III (yellow down triangle), NYHA IV (red diamond).
(DOCX)

S4 Fig Heart failure markers in pericardial fluid. The presence of miRNAs by NYHA grading for **a)** miR-320a, **b)** miR-22–3p, **c)** miR-92b-3p and **d)** miR-21–5p were measured using qPCR. Results are depicted as individual points for each measured sample (*n* = 51) lines indicating the overall mean for each group.
(DOCX)

S1 Table MicroRNA clusters in pericardial miRNome. (DOCX)

S2 Table MicroRNA families in pericardial miRNome. (DOCX)

S3 Table MicroRNA profiling results. (XLSX)
