# Supplementary figures and images for: MicroRNA Profiling of Pericardial Fluid Samples from Patients with Heart Failure
Source: PLoS One. 2015 Mar 12;10(3):e0119646. doi: 10.1371/journal.pone.0119646 (PMC4357463; doi:10.1371/journal.pone.0119646)

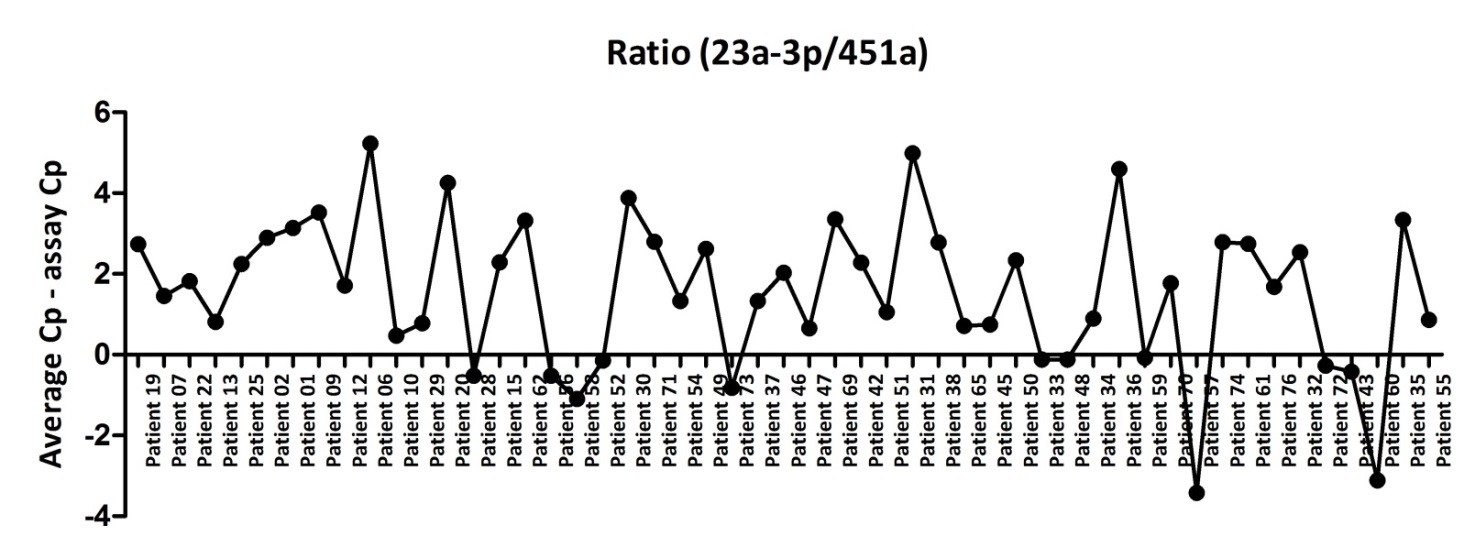


**Figure S1. Haemolysis.**

Supplement: S1 Fig — The ratio of two miRNAs is used to monitor haemolysis. MiR-451a is highly expressed in red blood cells, whereas miR-23a-3p is stably expressed and not affected by haemolysis. Samples with ratios above 8.0 are indicative of haemolysis. (DOCX) [file pone.0119646.s001.docx]

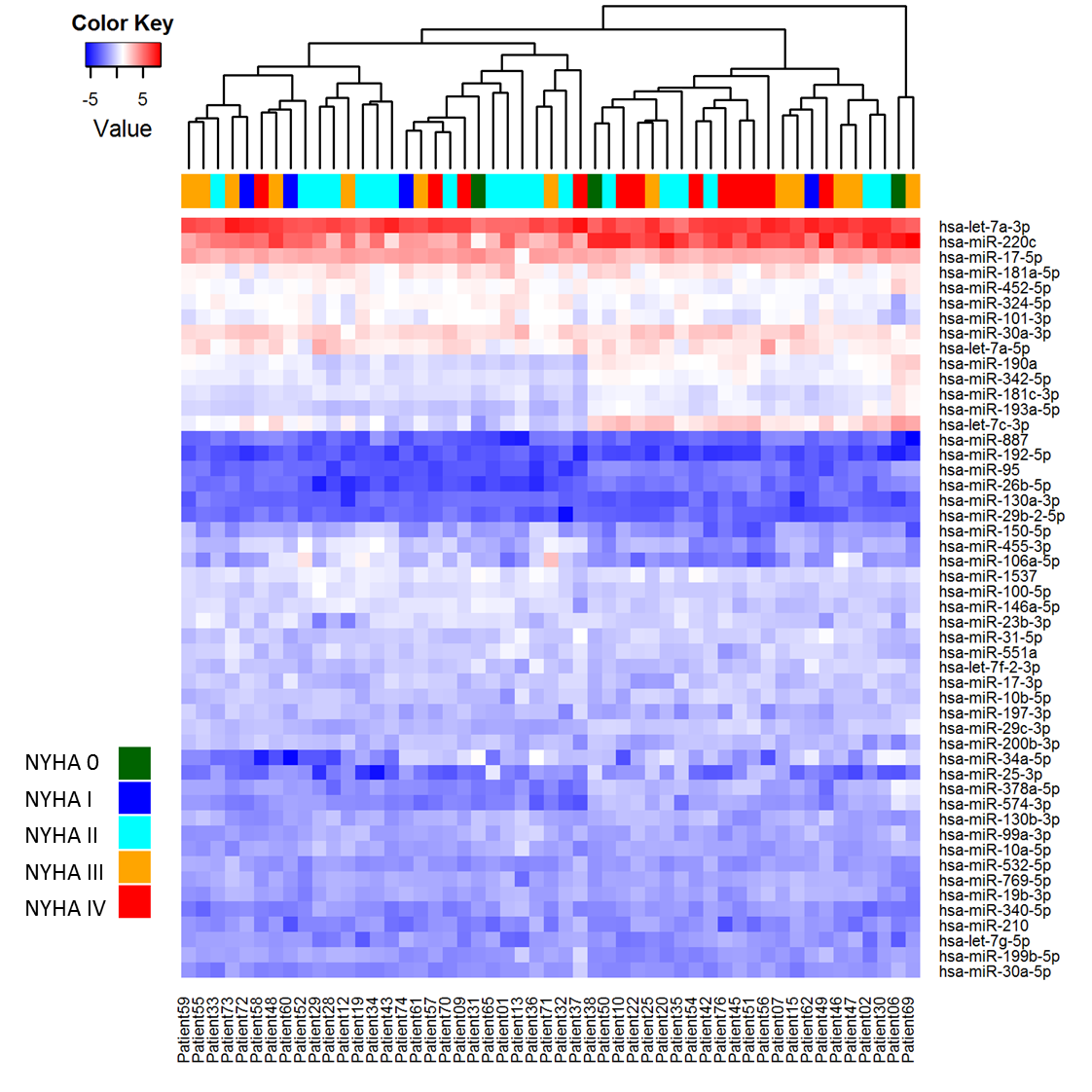


**Figure S2. Heat map and unsupervised hierarchical clustering**.

Supplement: S2 Fig — The clustering was performed on all samples and on the top 50 miRNAs with highest standard deviation. The normalised (dCp) values were used for the analysis. The colour scale illustrates the relative expression level of microRNA across all samples: red colour represents an expression level above mean, blue colour lower than the mean. The sample groups are colour-coded according to their NYHA grading: NYHA 0 (dark green), NYHA I (blue), NYHA II (cyan), NYHA III (orange), NYHA IV (red). (DOCX) [file pone.0119646.s002.docx]

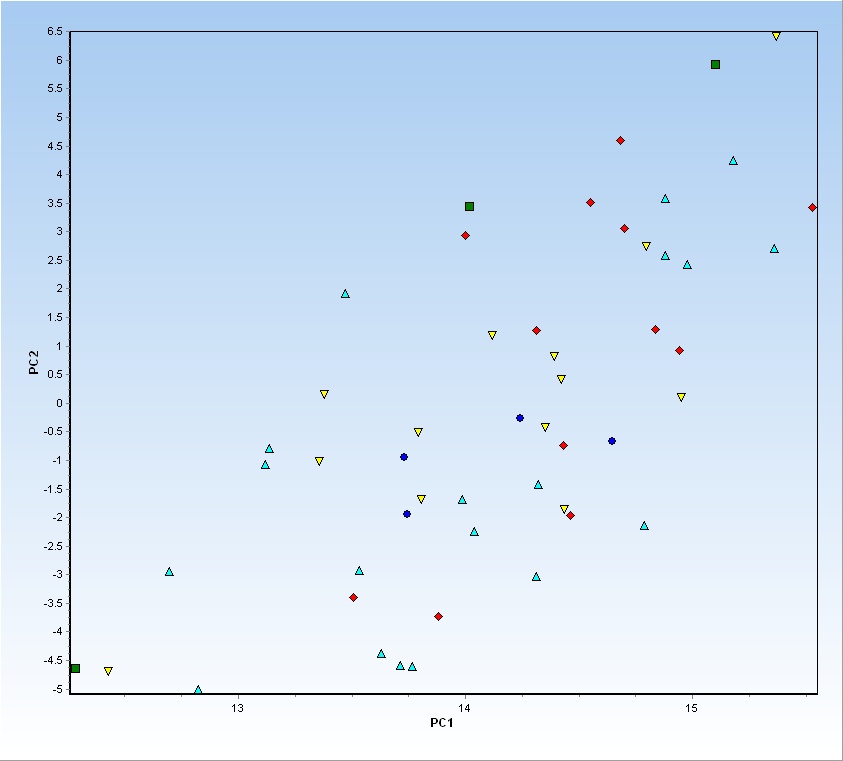


**Figure S3. Principal component analysis.**

Supplement: S3 Fig — The analysis is performed on all samples and on the top 50 miRNAs with highest standard deviation. The normalized (dCp) values were used for the analysis. Dots represent individual patients and colours indicate NYHA grading: NYHA 0 (green rectangle), NYHA I (blue circle), NYHA II (aqua triangle), NYHA III (yellow down triangle), NYHA IV (red diamond). (DOCX) [file pone.0119646.s003.docx]

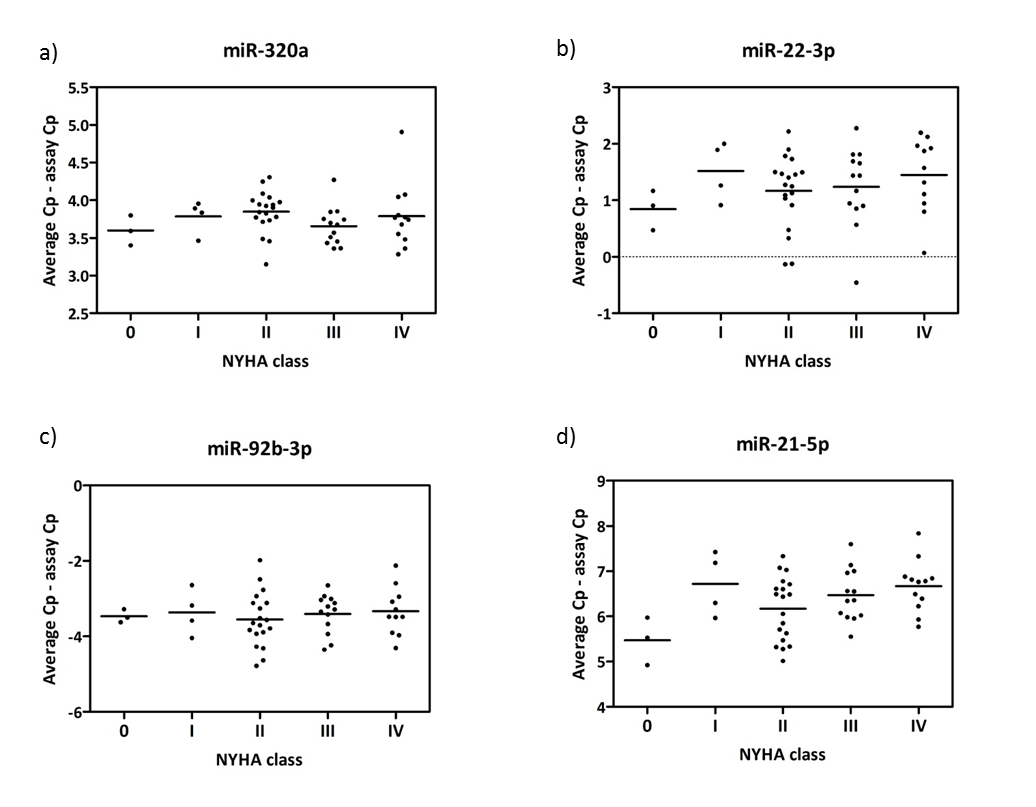


**Figure S4. Heart failure markers in pericardial fluid.**

Supplement: S4 Fig — The presence of miRNAs by NYHA grading for a) miR-320a, b) miR-22–3p, c) miR-92b-3p and d) miR-21–5p were measured using qPCR. Results are depicted as individual points for each measured sample (n = 51) lines indicating the overall mean for each group. (DOCX) [file pone.0119646.s004.docx]
