# Supplementary material for: MicroRNA Profiling of Pericardial Fluid Samples from Patients with Heart Failure
Source: PLoS One. 2015 Mar 12;10(3):e0119646. doi: 10.1371/journal.pone.0119646 (PMC4357463; doi:10.1371/journal.pone.0119646)
Supplement: S1 Table — (DOCX) [file pone.0119646.s005.docx]

**Table S1. MicroRNA clusters in pericardial miRNome**

| miRNA | Family | Cluster |
| --- | --- | --- |
| hsa-let-7a-3p | let-7 | hsa-let-7a-1, hsa-let-7f-1, hsa-let-7d /  hsa-let-7a-3, hsa-mir-4763, hsa-let-7b |
| hsa-let-7d-3p | let-7 | hsa-let-7a-1, hsa-let-7f-1, hsa-let-7d |
| hsa-let-7f-1-3p | let-7 | hsa-let-7a-1, hsa-let-7f-1, hsa-let-7d |
| hsa-let-7d-5p | let-7 | hsa-let-7a-1, hsa-let-7f-1, hsa-let-7d |
| hsa-let-7f-5p | let-7 | hsa-let-7a-1, hsa-let-7f-1, hsa-let-7d /  hsa-let-7f-2, hsa-mir-98 |
| hsa-let-7f-2-3p | let-7 | hsa-let-7f-2, hsa-mir-98 |
| hsa-let-7a-5p | let-7 | hsa-let-7a-1, hsa-let-7f-1, hsa-let-7d /  hsa-mir-100, hsa-let-7a-2 /  hsa-let-7a-3, hsa-mir-4763, hsa-let-7b |
| hsa-miR-100-5p | mir-10 | hsa-mir-100, hsa-let-7a-2 |
| hsa-let-7b-5p | let-7 | hsa-let-7a-3, hsa-mir-4763, hsa-let-7b |
| hsa-let-7b-3p | let-7 | hsa-let-7a-3, hsa-mir-4763, hsa-let-7b |
| hsa-miR-103a-2-5p | mir-103 | hsa-mir-103a-2, hsa-mir-103b-2 |
| hsa-miR-103a-3p | mir-103 | hsa-mir-103a-2, hsa-mir-103b-2 |
| hsa-miR-106a-5p | mir-17 | hsa-mir-106a, hsa-mir-18b, hsa-mir-20b, hsa-mir-19b-2,  hsa-mir-92a-2, hsa-mir-363 |
| hsa-miR-363-3p | mir-363 | hsa-mir-106a, hsa-mir-18b, hsa-mir-20b, hsa-mir-19b-2,  hsa-mir-92a-2, hsa-mir-363 |
| hsa-miR-19b-3p | mir-19 | hsa-mir-106a, hsa-mir-18b, hsa-mir-20b, hsa-mir-19b-2,  hsa-mir-92a-2, hsa-mir-363 /  hsa-mir-17, hsa-mir-18a, hsa-mir-19a, hsa-mir-20a, hsa-mir-19b-1,  hsa-mir-92a-1 |
| hsa-miR-20a-5p | mir-17 | hsa-mir-17, hsa-mir-18a, hsa-mir-19a, hsa-mir-20a, hsa-mir-19b-1,  hsa-mir-92a-1 |
| hsa-miR-17-3p | mir-17 | hsa-mir-17, hsa-mir-18a, hsa-mir-19a, hsa-mir-20a, hsa-mir-19b-1,  hsa-mir-92a-1 |
| hsa-miR-17-5p | mir-17 | hsa-mir-17, hsa-mir-18a, hsa-mir-19a, hsa-mir-20a, hsa-mir-19b-1,  hsa-mir-92a-1 |
| hsa-miR-92a-3p | mir-25 | hsa-mir-17, hsa-mir-18a, hsa-mir-19a, hsa-mir-20a, hsa-mir-19b-1,  hsa-mir-92a-1 |
| hsa-miR-93-5p | mir-17 | hsa-mir-106b, hsa-mir-93, hsa-mir-25 |
| hsa-miR-25-3p | mir-25 | hsa-mir-106b, hsa-mir-93, hsa-mir-25 |
| hsa-miR-143-3p | mir-143 | hsa-mir-143, hsa-mir-145 |
| hsa-miR-145-5p | mir-145 | hsa-mir-143, hsa-mir-145 |
| hsa-miR-342-3p | mir-342 | hsa-mir-151b, hsa-mir-342 |
| hsa-miR-342-5p | mir-342 | hsa-mir-151b, hsa-mir-342 |
| hsa-miR-16-5p | mir-15 | hsa-mir-15a, hsa-mir-16-1 |
| hsa-miR-15a-5p | mir-15 | hsa-mir-15a, hsa-mir-16-1 |
| hsa-miR-16-2-3p | mir-15 | hsa-mir-15b, hsa-mir-16-2 |
| hsa-miR-15b-5p | mir-15 | hsa-mir-15b, hsa-mir-16-2 |
| hsa-miR-15b-3p | mir-15 | hsa-mir-15b, hsa-mir-16-2 |
| hsa-miR-181b-5p | mir-181 | hsa-mir-181a-1, hsa-mir-181b-1 |
| hsa-miR-181a-5p | mir-181 | hsa-mir-181a-1, hsa-mir-181b-1 /  hsa-mir-181a-2, hsa-mir-181b-2 |
| hsa-miR-181a-2-3p | mir-181 | hsa-mir-181a-2, hsa-mir-181b-2 |
| hsa-miR-181c-5p | mir-181 | hsa-mir-181c, hsa-mir-181d |
| hsa-miR-181c-3p | mir-181 | hsa-mir-181c, hsa-mir-181d |
| hsa-miR-660-5p | mir-188 | hsa-mir-188, hsa-mir-500a, hsa-mir-362, hsa-mir-501, hsa-mir-500b,  hsa-mir-660, hsa-mir-502 |
| hsa-miR-425-5p | mir-425 | hsa-mir-191, hsa-mir-425 |
| hsa-miR-425-3p | mir-425 | hsa-mir-191, hsa-mir-425 |
| hsa-miR-193b-3p | mir-193 | hsa-mir-193b, hsa-mir-365a |
| hsa-miR-365a-3p | mir-365 | hsa-mir-193b, hsa-mir-365a |
| hsa-miR-215 | mir-192 | hsa-mir-194-1, hsa-mir-215 |
| hsa-miR-194-5p | mir-194 | hsa-mir-194-1, hsa-mir-215 /  hsa-mir-6750, hsa-mir-194-2, hsa-mir-192 |
| hsa-miR-192-5p | mir-192 | hsa-mir-6750, hsa-mir-194-2, hsa-mir-192 |
| hsa-miR-214-5p | mir-214 | hsa-mir-199a-2, hsa-mir-3120, hsa-mir-214 |
| hsa-miR-199a-5p | mir-199 | hsa-mir-199a-2, hsa-mir-3120, hsa-mir-214 |
| hsa-miR-200a-3p | mir-8 | hsa-mir-200b, hsa-mir-200a, hsa-mir-429 |
| hsa-miR-200b-3p | mir-8 | hsa-mir-200b, hsa-mir-200a, hsa-mir-429 |
| hsa-miR-141-3p | mir-8 | hsa-mir-200c, hsa-mir-141 |
| hsa-miR-200c-3p | mir-8 | hsa-mir-200c, hsa-mir-141 |
| hsa-miR-221-3p | mir-221 | hsa-mir-222, hsa-mir-221 |
| hsa-miR-222-3p | mir-221 | hsa-mir-222, hsa-mir-221 |
| hsa-miR-23a-3p | mir-23 | hsa-mir-23a, hsa-mir-27a, hsa-mir-24-2 |
| hsa-miR-24-2-5p | mir-24 | hsa-mir-23a, hsa-mir-27a, hsa-mir-24-2 |
| hsa-miR-27a-3p | mir-27 | hsa-mir-23a, hsa-mir-27a, hsa-mir-24-2 |
| hsa-miR-24-3p | mir-24 | hsa-mir-23a, hsa-mir-27a, hsa-mir-24-2 /  hsa-mir-23b, hsa-mir-27b, hsa-mir-3074, hsa-mir-24-1 |
| hsa-miR-23b-3p | mir-23 | hsa-mir-23b, hsa-mir-27b, hsa-mir-3074, hsa-mir-24-1 |
| hsa-miR-27b-3p | mir-27 | hsa-mir-23b, hsa-mir-27b, hsa-mir-3074, hsa-mir-24-1 |
| hsa-miR-29a-5p | mir-29 | hsa-mir-29b-1, hsa-mir-29a |
| hsa-miR-29a-3p | mir-29 | hsa-mir-29b-1, hsa-mir-29a |
| hsa-miR-29b-3p | mir-29 | hsa-mir-29b-1, hsa-mir-29a /  hsa-mir-29b-2, hsa-mir-29c |
| hsa-miR-29c-5p | mir-29 | hsa-mir-29b-2, hsa-mir-29c |
| hsa-miR-29c-3p | mir-29 | hsa-mir-29b-2, hsa-mir-29c |
| hsa-miR-29b-2-5p | mir-29 | hsa-mir-29b-2, hsa-mir-29c |
| hsa-miR-30b-5p | mir-30 | hsa-mir-30d, hsa-mir-30b |
| hsa-miR-30d-5p | mir-30 | hsa-mir-30d, hsa-mir-30b |
| hsa-miR-30d-3p | mir-30 | hsa-mir-30d, hsa-mir-30b |
| hsa-miR-30e-5p | mir-30 | hsa-mir-30e, hsa-mir-30c-1 |
| hsa-miR-30e-3p | mir-30 | hsa-mir-30e, hsa-mir-30c-1 |
| hsa-miR-30c-5p | mir-30 | hsa-mir-30e, hsa-mir-30c-1 |
| hsa-miR-374b-5p | mir-374 | hsa-mir-374c, hsa-mir-374b, hsa-mir-421 |
| hsa-miR-421 | mir-95 | hsa-mir-374c, hsa-mir-374b, hsa-mir-421 |
| hsa-miR-423-5p | mir-423 | hsa-mir-423, hsa-mir-3184 |
| hsa-miR-423-3p | mir-423 | hsa-mir-423, hsa-mir-3184 |
| hsa-miR-144-3p | mir-144 | hsa-mir-4732, hsa, mir-144, hsa-mir-451b, hsa-mir-451a |
| hsa-miR-451a | mir-451 | hsa-mir-4732, hsa, mir-144, hsa-mir-451b, hsa-mir-451a |
| hsa-miR-142-5p | mir-142 | hsa-mir-4736, hsa-mir-142 |
| hsa-miR-142-3p | mir-142 | hsa-mir-4736, hsa-mir-142 |
| hsa-miR-195-5p | mir-15 | hsa-mir-497, hsa-mir-195 |
| hsa-miR-497-5p | mir-497 | hsa-mir-497, hsa-mir-195 |
| hsa-miR-502-3p | mir-500 | hsa-mir-500a, hsa-mir-362, hsa-mir-501, hsa-mir-500b, hsa-mir-660,  hsa-mir-502 |
| hsa-miR-532-3p | mir-188 | hsa-mir-532, hsa-mir-188, hsa-mir-500a, hsa-mir-362, hsa-mir-501,  hsa-mir-500b |
| hsa-miR-532-5p | mir-188 | hsa-mir-532, hsa-mir-188, hsa-mir-500a, hsa-mir-362, hsa-mir-501,  hsa-mir-500b |
| hsa-miR-500a-5p | mir-500 | hsa-mir-532, hsa-mir-188, hsa-mir-500a, hsa-mir-362, hsa-mir-501,  hsa-mir-500b, hsa-mir-660, hsa-mir-502 |
| hsa-miR-501-3p | mir-500 | hsa-mir-532, hsa-mir-188, hsa-mir-500a, hsa-mir-362, hsa-mir-501,  hsa-mir-500b, hsa-mir-660, hsa-mir-502 |
| hsa-let-7c | let-7 | hsa-mir-99a, hsa-let-7c |
| hsa-miR-99a-3p | mir-10 | hsa-mir-99a, hsa-let-7c |
| hsa-miR-99a-5p | mir-10 | hsa-mir-99a, hsa-let-7c |
| hsa-let-7e-5p | let-7 | hsa-mir-99b, hsa-let-7e, hsa-mir-125a |
| hsa-miR-125a-5p | mir-10 | hsa-mir-99b, hsa-let-7e, hsa-mir-125a |
| hsa-miR-99b-5p | mir-10 | hsa-mir-99b, hsa-let-7e, hsa-mir-125a |
| hsa-miR-99b-3p | mir-10 | hsa-mir-99b, hsa-let-7e, hsa-mir-125a |
